# Transnasal Humidified Rapid Insufflation Ventilatory Exchange With Nasopharyngeal Airway Facilitates Apneic Oxygenation: A Randomized Clinical Noninferiority Trial

**DOI:** 10.3389/fmed.2020.577891

**Published:** 2020-11-27

**Authors:** Lingke Chen, Liu Yang, Weitian Tian, Xiao Zhang, Yanhua Zhao, Lili Huang, Jie Tian, Jiaqiang Zhang, Jiangxia Wu, Weifeng Yu, Diansan Su

**Affiliations:** ^1^Department of Anaesthesiology, Renji Hospital, Shanghai Jiaotong University School of Medicine, Shanghai, China; ^2^Department of Anesthesiology and Perioperative Medicine, Henan Provincial People's Hospital, People's Hospital of Zhengzhou University, Zhengzhou, China

**Keywords:** apneic oxygenation, arterial blood gas, general anesthesia, nasopharyngeal airway, THRIVE

## Abstract

**Background:** Transnasal humidified rapid insufflation ventilatory exchange (THRIVE) was used to extend the safe apnea time. However, THRIVE is only effective in patients with airway opening. Nasopharyngeal airway (NPA) is a simple device that can help to keep airway opening. This study aimed to investigate the noninferiority of NPA to jaw thrust for airway opening during anesthesia-induced apnea.

**Methods:** This was a prospective randomized single-blinded noninferiority clinical trial on the use of THRIVE in patients with anesthesia-induced apnea. The participants were randomly allocated to receive NPA or jaw thrust. The primary outcomes were PaO_2_ and PaCO_2_ at 20 min after apnea, with noninferiority margin criteria of −6.67 and 0.67 kPa, respectively.

**Results:** A total of 123 patients completed the trial: 61 in the NPA group and 62 in the jaw thrust group. PaO_2_ at 20 min after apnea was 42.9 ± 14.0 kPa in the NPA group and 42.7 ± 13.6 kPa in the jaw thrust group. The difference between these two means was 0.25 kPa (95% CI, −3.87 to 4.37 kPa). Since the lower boundary of the 95% CI was > −6.67 kPa, noninferiority was established because higher PO_2_ is better. PaCO_2_ at 20 min after apnea was 10.74 ± 1.09 kPa in the NPA group and 10.54 ± 1.18 kPa in the jaw thrust group. The difference between the two means was 0.19 kPa (95% CI, −0.14 to 0.53 kPa). Since the upper boundary of the 95% CI was <0.67 kPa, noninferiority was established because lower PCO_2_ is better. No patient had a SpO_2_ < 90% during apnea.

**Conclusion:** When THRIVE was applied during anesthesia-induced apnea, NPA placement kept airway opening and was noninferior to jaw thrust in terms of its effects on PaO_2_ and PaCO_2_ at 20 min after apnea.

**Clinical Trial Registration:** ClinicalTrials.gov (NCT03741998).

## Introduction

Transnasal humidified rapid insufflation ventilatory exchange (THRIVE) refers to a treatment method of continuously providing patients with adjustable and relatively constant oxygen concentration (21–100%), temperature (31–37°C), and humidity with high flow (8–70 L/min) inhalation of gas through a nasal high-flow oxygen inhalation device. THRIVE was first introduced by Dr. Patel in 2015 and was demonstrated to extend the apnea time to up to 65 min (1). In American Society of Anaesthesiologists (ASA) physical status I or II patients undergoing laryngeal surgery, the mean apnea time was reported to be 22.5 min (2). None of the patients in these studies desaturated below 90% (1, 2). Other studies investigated the use of THRIVE for awake fiber-optic intubation (3), tracheal intubation or emergency critically ill patients (4), and patients with intracranial hemorrhage (5). A physiological study on apneic oxygenation during laryngeal surgery showed that the use of THRIVE enabled patients with mild systemic disease and body mass index (BMI) <30 to maintain good oxygenation with pH ≥7.13 for a period of 30 min (2).

The potential applications of THRIVE in airway management include preoxygenation, extension of the safe apnea time, and post-extubation (6, 7). THRIVE was also applied in many laryngeal stenosis surgeries or airways with a certain degree of obstruction (8–12). Moreover, many questions still remain unanswered. The potential effects of THRIVE in specific settings, such as obese patients, elderly patients, children, and those for rapid sequence induction/intubation, need to be considered and investigated (13). However, keeping the airway open is the pre-requirement for the application of THRIVE because oxygenation is impossible in a totally blocked airway. Therefore, jaw thrust needs to be maintained in order to keep airway opening during apnea (6). Nasopharyngeal airway (NPA) is a simple supraglottic device that can form a channel in the nasopharynx, play a supporting role to the collapsed soft tissue, relieve nasopharynx obstruction, and keep the upper airway opening.

We hypothesized that NPA would be noninferior to jaw thrust for airway opening in patients using THRIVE for oxygenation during anesthesia-induced apnea. Therefore, the aim of this randomized controlled study was to compare two different methods of oxygenation in terms of arterial oxygen partial pressure (PaO_2_) and arterial carbon dioxide partial pressure (PaCO_2_) at 20 min after apnea.

## Materials and Methods

### Study Design

This prospective randomized single-blinded noninferiority clinical trial was conducted at Renji Hospital, which is affiliated to Shanghai Jiaotong University School of Medicine. This study was approved by local ethics committees (RenJiH[2018]011) and was registered at ClinicalTrials.gov (NCT03741998) on Nov 15, 2018. All the investigators underwent training for the technique of THRIVE.

### Patients

Adult patients >18 years of age, with ASA physical status I or II and who were for tracheal intubation general anesthesia surgery, were enrolled in this study. All the patients provided written informed consent. The exclusion criteria were as follows: (1) coagulation disorders or a tendency for nose bleeding, (2) an episode/exacerbation of congestive heart failure that required a change in medication, diet, or hospitalization from any cause in the last 6 months, (3) severe aortic stenosis or mitral stenosis, (4) cardiac surgery involving thoracotomy (e.g., coronary artery bypass graft, valve replacement surgery) in the last 6 months, (5) acute myocardial infarction in the last 6 months, (6) acute arrhythmia, including both tachycardia and bradycardia, with hemodynamic instability, (7) diagnosed chronic obstructive pulmonary disease or other acute or chronic lung disease requiring supplemental chronic or intermittent oxygen therapy, (8) increased intracranial pressure, (9) ASA >II, (10) mouth, nose, or throat infection, (11) fever, defined as core body temperature >37.5°C, (12) pregnancy, breastfeeding, or positive pregnancy test, (13) emergency procedure, and (14) known or suspected difficult airway.

### Randomization, Masking, and Sample Size Estimation

The randomization sequence was generated by a biostatistician, who did not take part in data management and statistical analyses. The PROC PLAN program in SAS (version 9.0) was used to generate the sample randomization sequence using 1:1 allocation with block = 20 and length = 6. The results of the randomization were sealed in sequentially numbered envelopes. Consecutively recruited patients were assigned to the NPA group or the jaw thrust group. The patients were masked because the NPA was inserted after loss of consciousness. Using PASS 16.0 software, the sample size was calculated with a noninferiority PaO_2_ margin of −6.67 kPa (noninferiority tests for the difference between two means). With alpha = 0.05 and power = 0.85, we determined that 120 patients (60 in each group) would be needed. We set the targeted enrollment at 126 patients to take into account risks of protocol deviations and consent withdrawals. Thus, we planned to include 63 patients in each group.

### Protocol

All the patients received standard monitoring, including three-lead electrocardiogram and peripheral oxygen saturation (SpO_2_), radial arterial line placement for invasive blood pressure monitoring and extraction for blood gas, and peripheral venous line placement for injection of medicine. Arterial blood gases were obtained before preoxygenation and every 5 min after anesthesia induction. The blood gases were analyzed using an ABL800 FLEX (Radiometer Medical ApS, Aakandevej 21, DK-2700 Bronshoj, Denmark). All the patients received awake preoxygenation with Optiflow^TM^ THRIVE system (Fisher & Paykel Healthcare, Auckland, New Zealand) at 40 L/min and 100% oxygen for 3 min. Anesthesia was induced by target-controlled infusion (TCI) of propofol (target plasma concentration, 4 μg/ml). The other I.V. induction was performed with midazolam (0.02–0.05 mg/kg) and sufentanil (0.4–0.6 μg/kg). When the patient was unconscious, rocuronium (0.6 mg/kg) was administered to achieve neuromuscular block. At the onset of apnea, which was indicated by disappearance of chest movement, the plasma target concentration of propofol TCI was adjusted to 2.0–3.0 μg/ml to maintain the anesthesia depth and avoid the possibility of awareness. The oxygen flow was increased and maintained at 60 L/min until tracheal intubation was completed.

**Figure 1:**
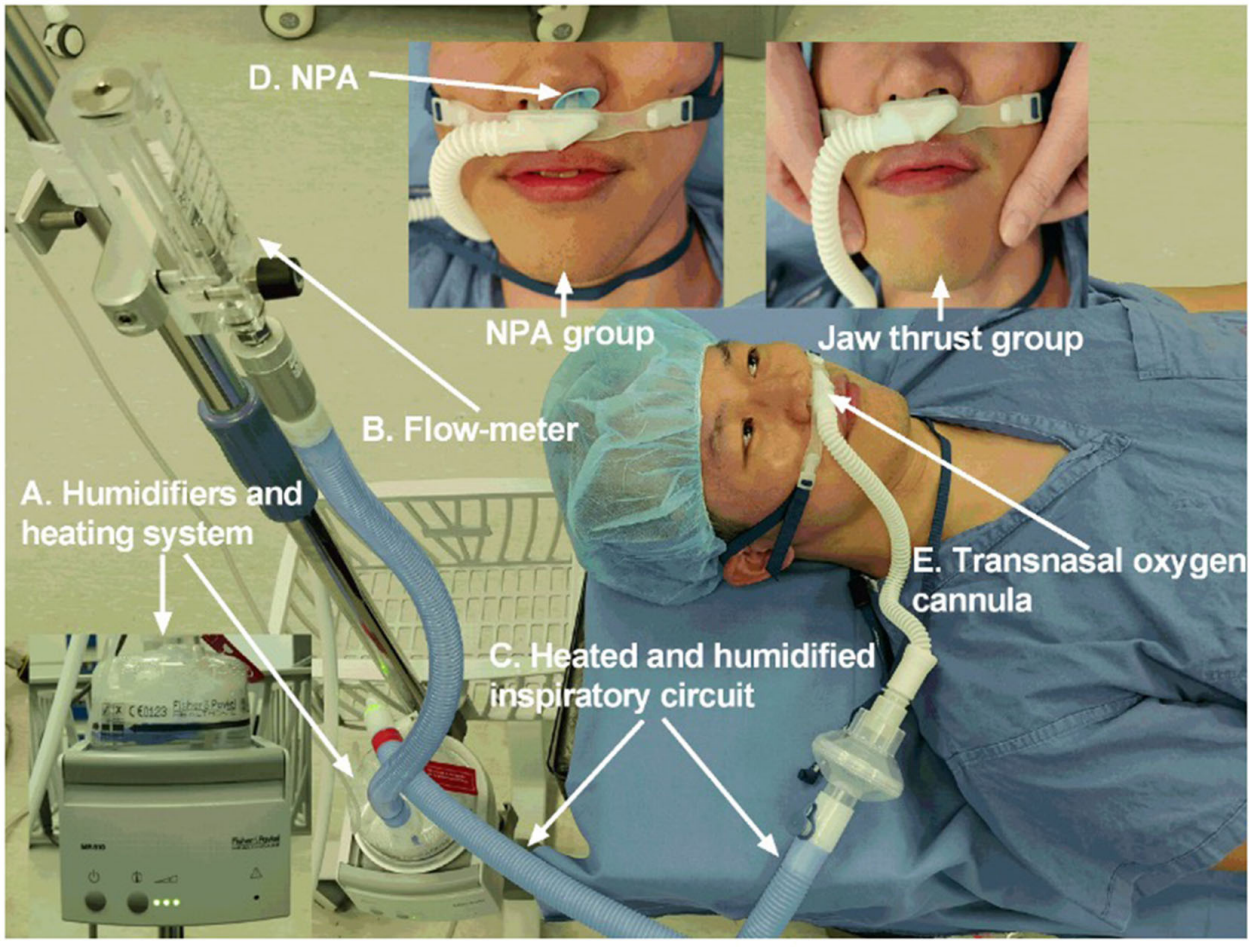
The NPA group and jaw thrust group received Optiflow^TM^ THRIVE system (Fisher & Paykel Healthcare, Auckland, New Zealand). **(A)** Humidifiers and heating system. **(B)** Flow meter. **(C)** Heated and humidified inspiratory circuit. **(D)** NPA. **(E)** Transnasal oxygen cannula. NPA, nasopharyngeal airway; THRIVE, transnasal humidified rapid insufflation ventilatory exchange.

THRIVE preoxygenation was the same for both study arms in the whole procedure. The only difference is that the NPA of appropriate type was placed after loss of consciousness in the NPA group, whereas patients allocated to the jaw thrust group were kept in jaw thrust to ensure upper airway opening. In the NPA group, high-flow nasal oxygen (HFNO) was administered over NPA (Figure 1).

When the systolic blood pressure was <90 mmHg, the plasma concentration of propofol TCI was decreased or ephedrine/phenylephrine was injected. When the heart rate (HR) fell below 50 bpm, atropine 0.5 mg was injected.

The patients in both groups were intubated under a video laryngoscope 20 min after anesthesia induction and were connected to a ventilator for machine-controlled ventilation. Oxygen flow was 2 L/min, oxygen concentration was 100%, tidal volume was 8–10 ml/kg and respiratory rate was 16 per minute. If the SpO_2_ decreased to 90% within 20 min, mask ventilation was applied immediately to correct the SpO_2_ to ≥95% before intubation. All operations (i.e., anesthesia induction, jaw thrust, NPA insertion, and tracheal intubation) were performed by the same senior anesthesiologist with more than 10 years of clinical experience.

SpO_2_, mean arterial pressure (MAP), HR, and arterial blood gas were recorded before preoxygenation, after preoxygenation, every 5 min after anesthesia induction, and at 10 min after intubation. Presence of nose bleeding was also recorded.

### Primary Outcomes

The primary outcomes were noninferior PaO_2_ and PaCO_2_ at 20 min after anesthesia induction and before intubation in the NPA group, compared with those in the jaw thrust group. The noninferiority margin was −6.67 kPa (−50 mmHg) for PaO_2_ and 0.67 kPa (5 mmHg) for PaCO_2_.

### Statistical Analysis

SPSS 23.0 (IBM, Armonk, NY, USA) was used for statistical analyses. Continuous variables were presented as means and standard deviations (SD) and compared using two-sample *t*-tests or Wilcoxon rank-sum tests as appropriate. Categorical variables were reported as frequencies and proportions and analyzed *via* chi-square or Fisher's exact test as appropriate. Values of *p* < 0.05 were considered statistically significant. The status of patient recruitment and dropout were summarized and listed. Demographic information and baseline characteristics, such as previous history of comorbidity, were recorded.

For noninferiority trials, choosing the noninferiority margin is a key and difficult issue. At present, there is no clear unified standard. Conventionally, the noninferiority margin is taken as the size of the effect considered clinically relevant (14). We set the noninferiority margins of PaO_2_ and PaCO_2_ at −6.67 and 0.67 kPa, respectively, which were half of the SD values based on data from our pilot study before this study started. These margins were set also after a discussion with a statistician and anesthesiology experts (14).

## Results

**Figure 2:**
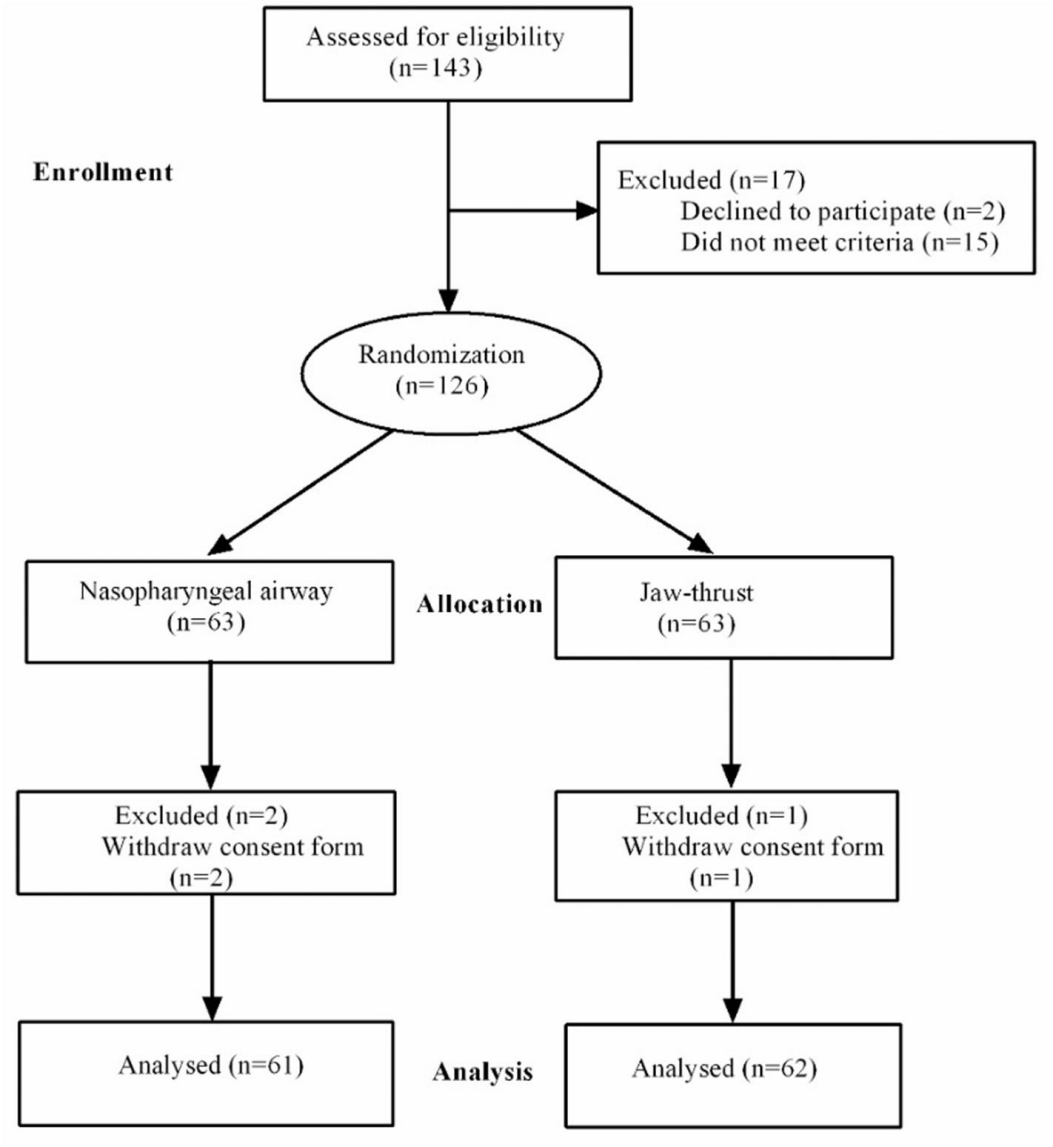
The CONSORT flow diagram.

A total of 143 patients were enrolled in the study. The patients were recruited between November 2018 and December 2019, with a final follow-up on December 1, 2019. There were 17 patients excluded: two patients declined to participate and 15 patients did not meet the criteria. In total, 126 patients were randomized into two groups as follows: NPA group (*n* = 63) and jaw thrust group (*n* = 63). The consent form was withdrawn by two patients in the NPA group and by one patient in the jaw thrust group. Finally, 61 patients in the NPA group and 62 patients in the jaw thrust group completed the trial and were included in the analyses (Figure 2).

Patient characteristics are reported in Table 1. There were no significant differences between the two groups in terms of sex, age, BMI, operation duration, post-anesthesia care unit stay duration, and hospital stay after surgery. Nasal bleeding was found in four patients (6.56%) in the NPA group and in one patient (1.61%) in the jaw thrust group. No patient had nasal bleeding during and after surgery.

**Table 1:** Demographic details and follow-up results of the participants undergoing transnasal humidified rapid insufflation ventilatory exchange oxygenation.

**Variable**	**Jaw thrust group (*n* = 62)**	**Nasopharyngeal airway group (*n* = 61)**	***P*-value**
Male, %	27 (43.5%)	32 (52.5%)	0.3227
Age, year	58.68 (16.58)	57.05 (16.49)	0.5860
Weight, kg	62.73 (11.13)	63.34 (11.09)	0.7582
BMI	23.10 (3.49)	23.27 (3.35)	0.7811
Nasal bleeding, %	1 (1.61)	4 (6.56)	
Duration of surgery, h	2.37 (1.15)	2.37 (1.79)	0.9974
Estimated blood loss, ml	136 (190)	115 (156)	0.4932
Length of PACU stay, min	76.94 (17.44)	76.34 (19.18)	0.8668
Length of hospital stay after surgery, days	7.18 (5.41)	6.36 (5.49)	0.4075

*Data are number (percentage) or mean ± standard deviation*.

*BMI, body mass index; PACU, post-anesthesia care unit*.

**Figure 3:**
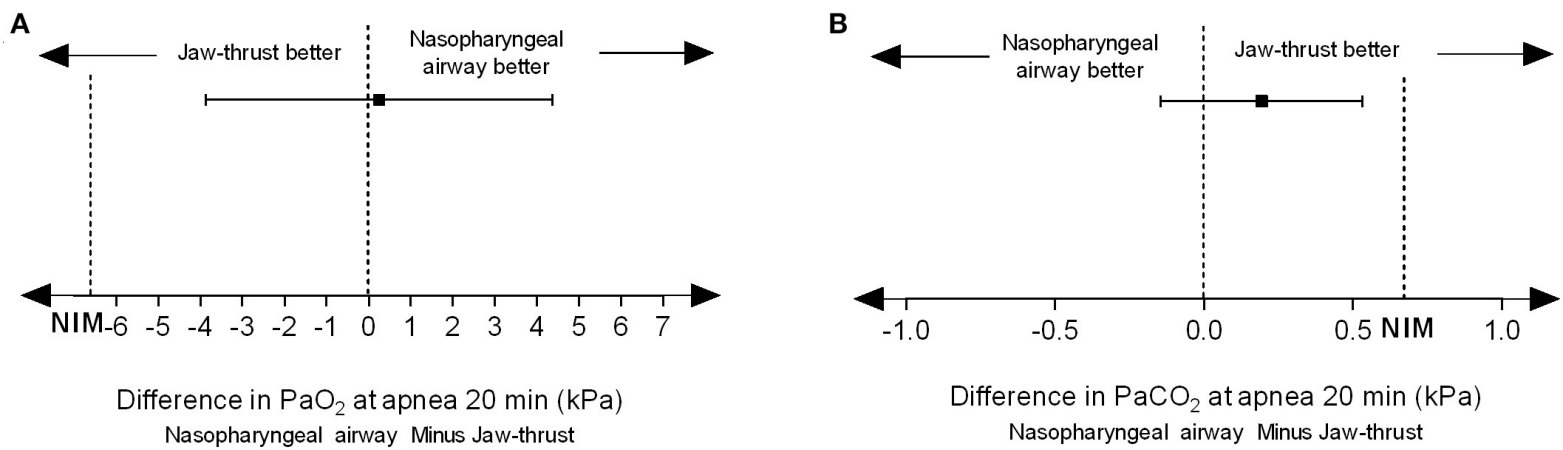
Nasopharyngeal airway facilitates transnasal humidified rapid insufflation ventilatory exchange and is noninferior to jaw thrust. **(A)** For PaO_2_, the difference value between the two groups was 0.25 kPa, and the 95% confidence interval was −3.87 to 4.37 kPa. Since the lower boundary of the confidence interval was > −6.67 kPa, noninferiority could be established because higher PO_2_ is better. **(B)** For PCO_2_, the difference value between the two groups was 0.19 kPa, and the 95% confidence interval was −0.14 to 0.53 kPa. Since the upper limit of the confidence interval was <0.67 kPa, noninferiority was established because lower PCO_2_ is better.

PaO_2_ at 20 min after apnea was 42.9 ± 14.0 kPa in the NPA group and 42.7 ± 13.6 kPa in the jaw thrust group. The difference between these two means was 0.25 kPa (95% CI, −3.87 to 4.37 kPa). Since the lower boundary of the 95% CI was within the noninferiority margin of −6.67 kPa, noninferiority was established because higher PO_2_ is better (Figure 3A). PaCO_2_ at 20 min after apnea was 10.74 ± 1.09 kPa in the NPA group and 10.54 ± 1.18 kPa in the jaw thrust group. The difference between the two means was 0.19 kPa (95% CI, −0.14 to 0.53 kPa). Since the upper boundary of the confidence interval was <0.67 kPa, noninferiority was established because lower PCO_2_ is better (Figure 3B).

**Figure 4:**
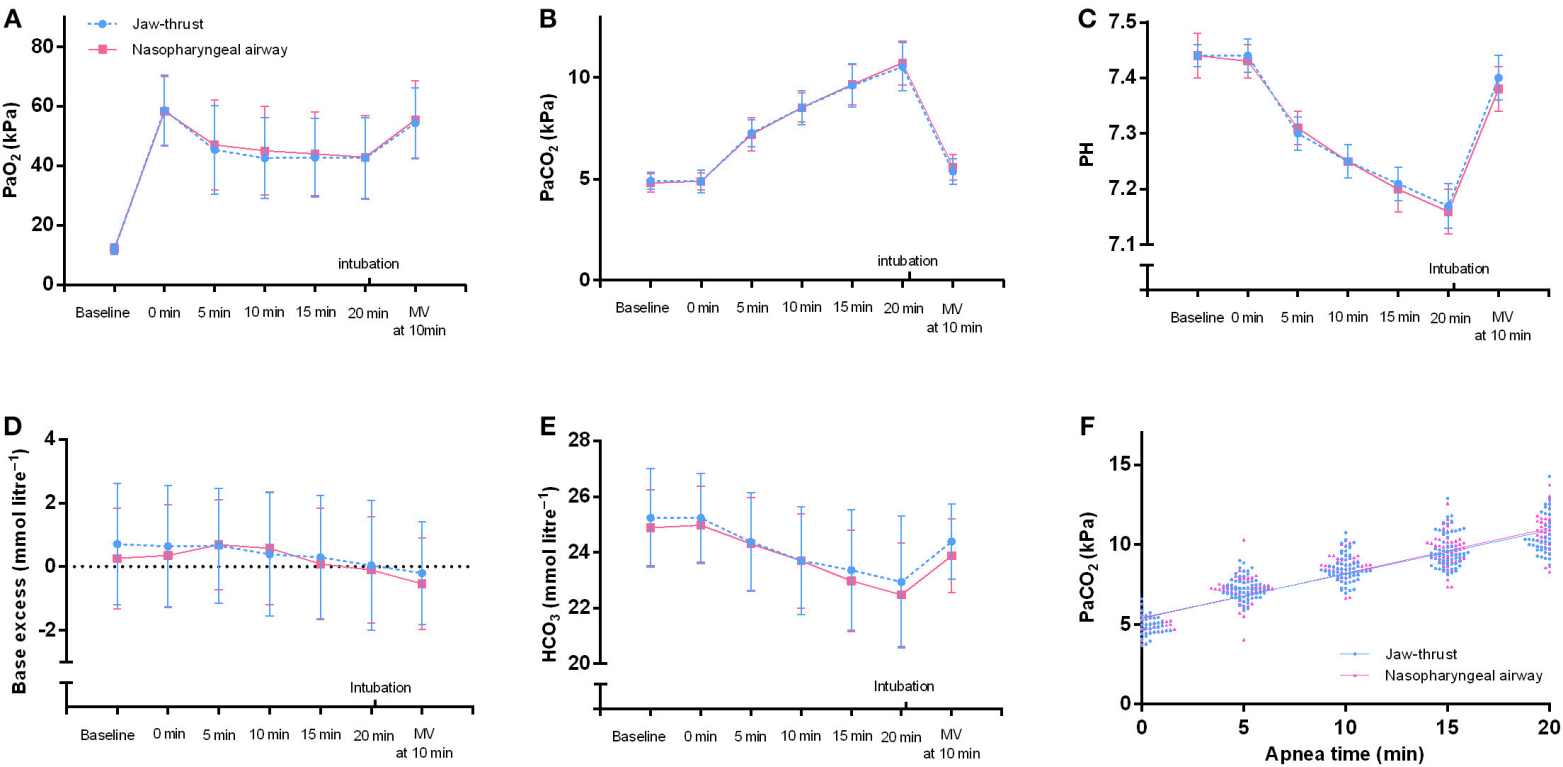
Arterial blood gases of the nasopharyngeal airway and jaw thrust groups during transnasal humidified rapid insufflation ventilatory exchange. There are no significant differences in PaO_2_ **(A)**, PaCO_2_ **(B)**, pH **(C)**, standard base excess **(D)**, and HCO_3_^−^ **(E)** between the two groups. **(F)** For the nasopharyngeal airway group, the line represents linear regression with *r* = 0.8324 and *p* < 0.0001. The regression equation was *Y* = 2.123 *X + 40.30. For the jaw thrust group, the line represents linear regression with *r* = 0.7999 and *p* < 0.0001. The regression equation was *Y* = 2.054 *X + 40.68. MV, mechanical ventilation.

At every time point, the PaO_2_ and the PaCO_2_ did not significantly differ between the NPA and the jaw thrust groups (Figures 4A,B). Similarly, no significant difference was found in other arterial blood gas parameters of pH, ${\text{HCO}}_{3}^{-}$, and standard base excess between the two groups (Figures 4C–E). With the extension of apnea, the PaCO_2_ increased in a linear pattern (*r* = 0.83, *p* < 0.0001 in the NPA group; *r* = 0.80, *p* < 0.0001 in the jaw thrust group) (Figure 4F).

**Figure 5:**
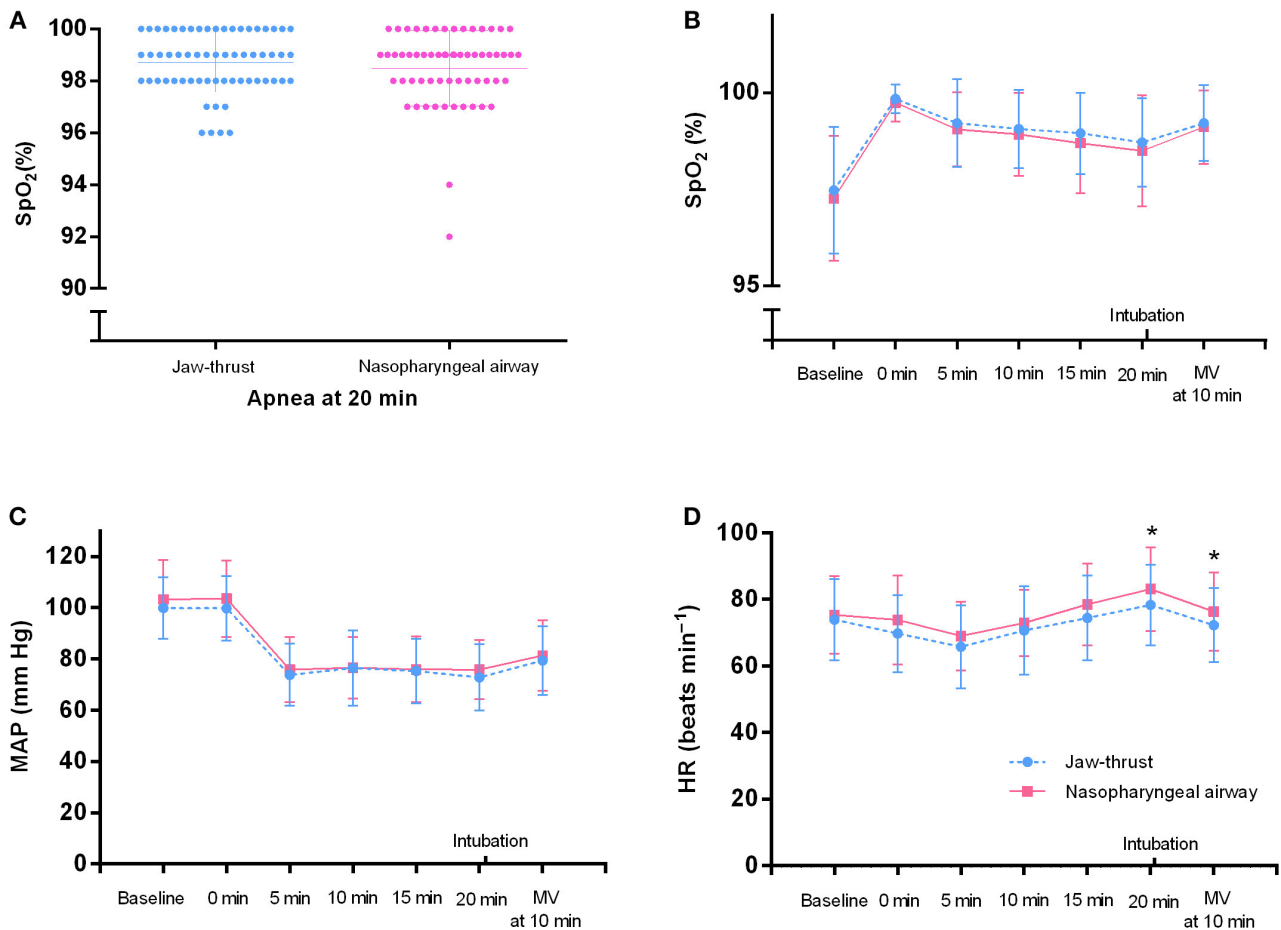
Vital signs of the nasopharyngeal airway (NPA) and jaw thrust groups during transnasal humidified rapid insufflation ventilatory exchange. **(A)** In all patients, the SpO_2_ at 20 min after apnea is >90%. **(B,C)** There are no significant differences between the two groups for the SpO_2_ and mean arterial pressure (MAP) at each time point. **(D)** At 20 min after apnea and 10 min after intubation, the heart rate (HR) is higher in the NPA group than in the jaw thrust group. There are no significant differences between the two groups at the other time points. MV, mechanical ventilation.

At 20 min after apnea, the SpO_2_ in all patients was >90% (Figure 5A). No significant differences were found between the two groups for the SpO_2_ and the MAP at each time point (Figures 5B,C). No significant differences were found between the two groups for the HR before preoxygenation (baseline), after preoxygenation (0 min), and at 5, 10, and 15 min during apnea (Figure 5D). Notably, compared with the jaw thrust group, the NPA group had higher HR at the time points of apnea 20 min (83.13 ± 12.58 vs. 78.35 ± 12.10 bpm) and 10 min after mechanical ventilation (76.39 ± 11.76 vs. 72.29 ± 11.14 bpm) (Figure 5D), although this had no clinical significance. Nose bleeding was found in only four patients (6.56%) with no harm in the NPA group and did not need further treatment.

## Discussion

HFNO has been applied during both spontaneous breathing and apnea (15). Recently, THRIVE was also reported to prolong the safe apnea time. Keeping the upper airway open is the prerequisite to ensure that THRIVE works well. When deep sedation or neuromuscular blocking drugs are used, jaw thrust has to be done to ensure upper airway opening (6). The present randomized clinical trial demonstrated that NPA was able to facilitate THRIVE. The two key parameters, PaO_2_ and PaCO_2_, were noninferior to jaw thrust. THRIVE is especially valuable in cases of difficult airway by extending the apnea time up to 65 min (1).

In the present study, no patient had an SpO_2_ <90% during apnea. Similar to another study, the major limiting factor of the extension of apneic oxygenation is the increase of CO_2_ but not oxygenation (2). The rates of PaCO_2_ increase varied widely among different studies. The diffusion of CO_2_ from the alveoli during apnea depends on the airway opening. After mimicking total airway occlusion and no CO_2_ washout, Stock (16) demonstrated an increase in CO_2_ rate of 0.68 kPa/min. Gustafsson demonstrated an increase in the rate of PaCO_2_ of 0.24 kPa/min during the apnea duration in patients undergoing shorter laryngeal surgery (2). In the study of Dr. Patel, the rate of CO_2_ increase was 0.15 kPa/min during the apnea duration (1). In this study, the increase in the rate of CO_2_ was 0.28 kPa/min in the jaw thrust group and 0.29 kPa/min in the NPA group; these were higher compared with those in the study of Dr. Patel (1). The present study measured the rate of CO_2_ increase according to the blood gas PaCO_2_ value but not capnography. Gustafsson (2) demonstrated that the difference between ETCO_2_ and PaCO_2_ increased significantly with the extension of apnea time; therefore, capnography is clearly not a reliable method of monitoring during extended apnea. Of course, capnography cannot be used during apneic oxygenation (2). The increased rate of PaCO_2_ was similar between the present study and the Gustafsson (2) study (0.24 kPa/min), both of which used PaCO_2_ for calculation. Muscular activation would affect the production of CO_2_ from the skeletal muscle (17). In this study, no additional neuromuscular blocking drug was administered; therefore, the minimal recovery of neuromuscular function during apnea might have affected the CO_2_ clearance, which would help explain the little-bit-higher increased CO_2_ rate in the present study than in the Gustafsson study. Similar to Dr. Patel's study (1), this study showed a linear pattern of PaCO_2_ increase with apnea time in both groups.

HFNO or THRIVE has been used in several anesthesia settings. Miguel-Montane (4) demonstrated that HFNO decreased the hypoxia rate during tracheal intubation of intensive care patients. Our previous study showed that HFNO significantly decreased the hypoxia rate during sedated gastroscopy procedure (18). Badiger (3) demonstrated that THRIVE reduced episodes of desaturation during awake fiber-optic intubation. The study of Mir (19) showed that THRIVE was a practical method for pre-oxygenating patients for rapid sequence induction. HFNO can also be used in patients with airway stenosis (8), such as subglottic stenosis (9–11) and tracheal stenosis caused by thyroid tumor (12).

NPA is one simple airway adjunct to help keep the airway open, regardless of the presence of spontaneous breathing. During tracheal intubation under spontaneous breathing, patients could have respiratory depression because of oversedation (20, 21). In this case, NPA is a much better choice than oropharyngeal airway because NPA does not cause gagging in patients. Moreover, a large proportion of patients who need “tracheal intubation under spontaneous breathing” do not have feasible oral access due to mouth trauma or other issues. In these patients, an oropharyngeal airway cannot or should not be used, and the NPA has obvious advantages. In the present study, placement of NPA during apnea was noninferior to jaw thrust, which would be greatly helpful to keep the airway open for severely obese patients for whom jaw thrust was often ineffective and difficult to maintain for a longer time.

Nasal bleeding is the main concern of NPA placement. Actually, in patients without hemorrhagic tendency, use of vasoconstrictors, relatively small size, good lubrication, and soft rubber NPA would help reduce the nose bleeding rate. In the present study, nose bleeding was found in only four patients (6.56%) in the NPA group, which did not need further treatment. None of the patients had nasal bleeding during and after surgery. In addition, in order to avoid aspiration secondary to nasopharyngeal bleeding, suction devices should be prepared before anesthesia induction.

The present study had several limitations. First, the apnea time was set to 20 min instead of a longer period. Therefore, we do not know whether the NPA would remain noninferior to jaw thrust during THRIVE with apnea time longer than 20 min. Second, no obese patient was enrolled in the present study; therefore, more studies are warranted to prove the effects of the NPA when using THRIVE on obese patients. Third, no neuromuscular block monitor was used. A neuromuscular block monitor would help indicate the start point of apnea after induction. According to Jeung and Shahnawaz, 0.6 mg/kg of rocuronium plus the indicated doses of other induction anesthetics would terminate spontaneous breathing in a normal adult within 1 min (22, 23). Therefore, the performer, who is the same person who analyzed all the recruited patients, would keep a close eye on the patients' chest movement since the start of anesthetic induction to guarantee that the onset of apnea would be immediately detected. Even if some variations exist, it would be small. Therefore, similar with some other studies (19), we did not monitor neuromuscular block.

In conclusion, keeping the upper airway open is a prerequisite for the application of THRIVE. Compared with jaw thrust, the simple method of NPA placement to ensure upper airway opening during THRIVE had noninferior effects on PaO_2_ and PaCO_2_ at 20 min after apnea. Attention should be paid to nose bleeding caused by NPA placement.

## Data Availability Statement

The raw data supporting the conclusions of this article will be made available by the authors, without undue reservation.

## Ethics Statement

The studies involving human participants were reviewed and approved by Clinical Research Ethics Committee of Renji Hospital in the School of Medicine of Shanghai Jiaotong University. The patients/participants provided their written informed consent to participate in this study. Written informed consent was obtained from the individual(s) for the publication of any potentially identifiable images or data included in this article.

## Author Contributions

DS and LC participated in study concept and design, statistical analysis, and analysis and interpretation of data. DS, LY, and WY drafted the article. LC, LY, WT, XZ, YZ, LH, JT, JZ, JW, and WY performed the research and analyzed the data. All the authors interpreted the data, revised the drafts, and approved the final version.

## Conflict of Interest

The authors declare that the research was conducted in the absence of any commercial or financial relationships that could be construed as a potential conflict of interest.
